# LiCl *in situ* decorated metal–organic framework (MOF)-derived porous carbon for efficient solar-driven atmospheric water harvesting†

**DOI:** 10.1039/d4ra02364a

**Published:** 2024-05-14

**Authors:** Simiao Guo, Yue Hu, Zhou Fang, Bing Yao, Xinsheng Peng

**Affiliations:** a State Key Laboratory of Silicon and Advanced Semiconductor Materials, School of Materials Science and Engineering, Zhejiang University Hangzhou 310058 P. R. China pengxinsheng@zju.edu.cn; b Wenzhou Key Laboratory of Novel Optoelectronic and Nanomaterials, Institute of Wenzhou, Zhejiang University Wenzhou 325006 P. R. China

## Abstract

Solar-powered sorption-based atmospheric water harvesting (AWH) technology is a promising solution to the freshwater scarcity in arid regions. Existing adsorbent materials still face challenges in aspects such as cycling stability and adsorption kinetics and require further development. Herein, we presented a strategy for the *in situ* fabrication of high-performance adsorbents, lithium chloride (LiCl)-decorated metal–organic framework (MOF)-derived porous carbon sorbents (PCl), *via* high-temperature pyrolysis and hydrogen chloride (HCl) vapor treatment. The sorbents display high adsorption capacity across a wide range of humidity water adsorption capacities in a wide humidity range with the maximum adsorption capacity of 7.87 g g^−1^, and rapid response to the solar-driven process and excellent cyclic stability. The LiCl nanocrystals in PCl can be utilized efficiently and decorated within the porous framework stably, and demonstrate water adsorption at 20%, 40%, 60% and 80% RH, of 1.34, 1.69, 2.56 and 4.23 g_H_2_O_·g_LiCl_−1, respectively, and significantly higher water uptake and release rates than bulk LiCl. This may provide new guides for designing efficient solar-driven AWH.

## Abstract 2

LiCl *in situ* decorated MOF-derived porous carbon sorbents (PCl-4) were prepared from Li-MOFs for atmospheric water harvesting. PCl-4 displays excellent water-capture capabilities and efficient solar-driven water release with nice recyclability.

## Graphical abstract

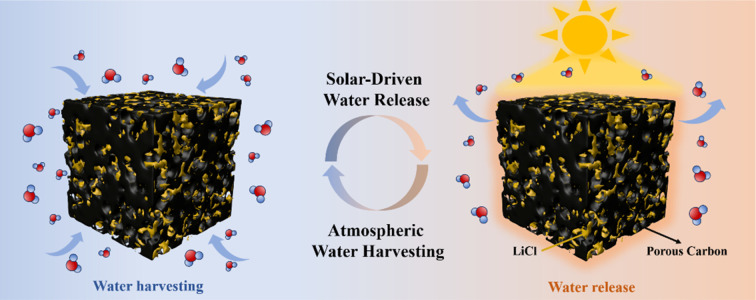

## Introduction

Water resources play a vital role in sustaining human life and facilitating development.^1^ The global challenge of freshwater scarcity has emerged due to environmental pollution and population expansion.^2,3^ There have been attempts to address water shortage problems through projects such as seawater desalination, wastewater reuse and building more dams and wells, but these efforts may not be enough, particularly in dry and hot regions and inland areas far from water resources.^4^ Alternatively, the water source in the air has been considered to meet the survival needs of humans living in arid and inland areas, which exists in the form of vapor and small droplets, with an estimated content of 1.3 × 10^13^ m^3^ approximately, equivalent to six times the volume of river water on Earth.^5^ Water collected from the air can meet drinking water standards without complex purification and disinfection. Furthermore, water collection from the atmosphere requires minimal centralized infrastructure and is not constrained by specific regions.^6^ Atmospheric water harvesting (AWH) has become a promising technique for water yield during the past decades, such as hydrophilic surface-enabled fog harvesting,^7^ dew-water collection,^8,9^ and sorption-based AWH, which are major strategies employed to achieve effective AWH. Among them, fog harvesting with limited efficiency, is highly only recommended for specific locations with high relative humidity (RH).^10,11^ The dew water collection requires lowering the air temperature below the dew point through condensation to collect water, making it applicable in a broader range. However, the energy consumption increases significantly, especially when the environmental temperature and humidity decrease.^12,13^ Currently, sorption-based AWH, which includes moisture sorbents to harvest water vapor from the air and thermal processes to release water, has emerged as a promising method for clean water generation.^14^ Meanwhile, the regeneration of sorbents and desorption of water could be achieved by using clean energy such as solar energy, which can work in arid areas (RH ≤ 30%) and lead to simple, affordable, and all-in-one devices.^15,16^

The properties of sorbents determine the upper limit of water production and adsorption kinetics.^17^ Traditional physical adsorbents such as silica gel and zeolite exhibit low water production and a high regeneration temperature (*T* > 100 °C).^18^ Hygroscopic salts including calcium chloride (CaCl_2_), lithium chloride (LiCl) can quickly absorb water molecules due to chemical hydration which lead to high water absorption capacity.^12,15,19^ However, the deliquescent properties and difficulty in water release of hygroscopic salts limit their application in atmospheric water collection.^14^ Metal–organic frameworks (MOFs) are widely used in gas transportation, separation, storage and catalysis. Numerous metal centers and organic ligands can endow MOFs with diverse structures and functions. The high porosity of MOFs provides abundant active sites for the adsorption of water molecules, making MOFs the most promising candidates for water adsorption applications.^20,21^ Unfortunately, the majority of them are suffering from rather slow atmospheric water adsorption kinetics due to the restricted diffusion of water molecules in their microporous matrices.^22,23^ And most MOFs have poor light absorption capabilities, making it difficult to reach high temperatures under sunlight and causing a low water release rate.^24^ Compared with MOFs, the excellent photothermal properties of porous carbons can enable rapid solar-driven desorption. However, they have attracted much less interest in AWH application due to the inherently low affinity between water and carbon.^25,26^

Therefore, it's essential to develop adsorbents materials with high adsorption capacity, fast adsorption–desorption kinetics, and excellent photothermal properties for solar-driven sorption-based AWH.^27^ To solve these problems, researchers try to incorporate hygroscopic salt with high water adsorption capacity into a porous carrier matrix to form a new salt-based composite material, in which the porous matrix is used to solve the agglomeration and solution leakage of salts.^28,29^ However, these composites still have challenges in terms of adsorption–desorption kinetics and cycling stability. In addition, a MOF-templated strategy was proposed, that is, synthesizing nanoporous carbon through the thermal transformation of MOF precursors.^30^ It has shown that MOF-derived microporous carbons were able to capture water vapor rapidly at low RH, mainly due to the presence of MOF precursor-derived hydrophilic heteroatoms.^25^ The carbonaceous sorbents demonstrate rapid desorption kinetics facilitated by effective solar-thermal heating and elevated thermal conductivity. Furthermore, an oxygen plasma-treated magnetic flower-like porous carbon (P-MFPC) with large open surfaces and abundant surface oxygen-containing moieties is developed,^26^ which exhibited extremely fast atmospheric water adsorption and water desorption kinetics by localized magnetic induction heating. Consequently, MOF-derived porous carbons hold great promise for achieving highly productive and energy-efficient AWH because of outstanding photothermal conversion properties and structural designability.^31^ However, these MOF-derived porous carbons have low adsorption capacity in desert climates and require multiple adsorption–desorption times to achieve high yield.

Considering that most existing salt-based composite sorbents impregnate hygroscopic salts in the porous matrix, we reported a novel method to develop photothermal MOF-derived sorbents in very recently.^24^ By converting stable calcium MOF into porous carbons (PC) *via* the pyrolysis process, followed by hydrogen chloride (HCl) vapor treatment, CaCl_2_-decorated porous carbon sorbents (PCC-42) were fabricated *in situ*. The resulting sorbents exhibited good adsorption performance across a wide range of humidity levels. However, the chemically adsorbed crystal water of CaCl_2_·2H_2_O begins to release above 200 °C and can only be completely desorbed when the temperature is greater than 250 °C.^32^ Limited by the high regeneration temperature, PCC-42 could not completely release water. Additionally, the inherent limited adsorption capacity of pure CaCl_2_ restricts the overall adsorption capacity of PCC-42. In comparison, the adsorption performance of LiCl is higher than that of CaCl_2_ and the desorption temperature of LiCl is lower (around 85 °C).^12^ These indicate that using LiCl as the adsorption sites has the potential to develop adsorbents with superior performance. Inspired by these, we report MOF-derived porous carbon sorbents with another hygroscopic salts LiCl decorated uniformly and excellent photothermal properties to achieve high-yield solar-driven AWH. LiCl has a higher adsorption capacity than that of CaCl_2_ and it is easier for LiCl to achieve rapid desorption, so we attempted to use LiCl as the adsorption sites to improve the overall adsorption capacity and desorption performance of the sorbents. We embarked on an exploration of frameworks based on lithium metal centers and choose lithium MOF (Li_2_(4, 4′-BPDC)) as the starting material. LiMOF was transformed into PC *via* high-temperature pyrolysis, followed by HCl vapor treatment^24^ to achieve LiCl-decorated porous sorbents (PCl) *in situ* with controllable LiCl content. LiMOF not only served as a carbonaceous precursor to produce porous carbons, but also ensured the well-distribution of lithium metal sources to form LiCl, endowing the resulting sorbents with adequate adsorption sites. The obtained adsorbents can effectively prevent salt leakage and agglomeration. LiCl, as the adsorption sites, has high water adsorption capacity over a wide humidity range. And porous carbons with nanoscale pores and high porosity, facilitate full contact between water molecules and adsorption sites. The photothermal performance of porous carbons could enable rapid heat transfer under sunlight, promoting the desorption of water molecules. The operation of the sorbents for AWH comprises two steps: water capture from ambient air and water release by solar-driven steam generation. By synergistically taking advantage of these merits, LiCl-decorated porous sorbents exhibit much improved sorption kinetics and cyclic stability compared to pure LiCl powders and porous carbons. Compared with our previous work, the overall adsorption capacity of this novel PCl sorbents is improved. Firstly, the highest water uptake capacity of PCC-42 is 3.04 g g^−1^, while PCl-4 can reach up to 7.87 g g^−1^. Moreover, the desorption rate is faster. The desorption rate of PCl-4 at 20% RH is 0.57 kg kg^−1^ h^−1^, while that of PCC-42 is only 0.38 kg kg^−1^ h^−1^. And the water release performance of PCl-4 reaches 88% within 1.5 hours (PCC-42 is around 80%). These further illustrates the generalizability of our *in situ* transformation design strategy. Finally, we demonstrated a lab-scale water harvester for applications achieving 0.83 L_water_ kg_adsorbents_^−1^ per cycle under RH% and standard one sun irradiation in 5.5 hours. The proposed design strategy is beneficial for the development of high-yield, solar-driven AWH systems in advanced freshwater-generation applications.

## Experimental

### Chemicals and materials

Lithium nitrate (LiNO_3_, 99.99%), 4,4′-biphenyl dicarboxylic acid (C_14_H_10_O_4_, 4,4′-BPDC, 97%), ammonium fluoride (NH_4_F, 99.99%) were obtained from Macklin. *N*,*N*′-dimethyl formamide (C_3_H_7_NO, DMF, AR), hydrochloric acid (HCl, AR) and ethanol (C_2_H_5_OH, AR) were purchased from Chengdu Chron Chemicals Co., Ltd. Deionized (DI) water (18.2 MΩ cm, from Milli-Q system) was used in all experiments. All the chemicals were used without further purification.

### Synthesis of the LiMOF, PC and PCl sorbents

LiMOF was synthesized according to the previous method with slight modifications.^33^ LiNO_3_ (3.75 mmol, 0.258 g), 4,4′-BPDC (3.75 mmol, 0.9084 g) and NH_4_F (1.5 mmol, 0.0555 g) were dissolved in DMF (27.4 mL) in a 100 mL Teflon container. Then, it was sonicated for 20 minutes to achieve homogeneity. The resultant solution was heated for 4 days at 180 °C. The product obtained was recovered by filtration, subsequently washed with ethanol for three times and finally dried at 120 °C for 12 hours.

For the preparation of PC samples, LiMOF was heated to a certain temperature (500 °C, 600 °C and 700 °C) in a tube furnace at a rate of 10 °C min^−1^ under nitrogen condition and maintained for 2 hours. Then it was cooled to room temperature, called PC-500, PC-600, and PC-700, respectively.

For the preparation of PCl-*x*, about 30 mg PC-700 was placed in a container with 20 mL HCl (36–38%) vapor treatment for certain hours and dried at 120 °C in oven overnight. The obtained samples were named as PCl-*x*, where *x* was 1, 4, 12 hours corresponding to the treatment time of HCl vapor.

### Water sorption

The water adsorption properties of all samples were tested at 25 °C and under different relative humidity (20% RH, 40% RH, 60% RH, 80% RH). All samples were dried at 120 °C in oven overnight before each adsorption test and then placed into a jar for water adsorption under the above conditions. They were weighed at regular intervals using an analytical balance with an accuracy of 0.0001 g, and then exposed to simulated sunlight at 1 kW m^−2^ for desorption.

The cycling stability tests of PCl-4 were conducted under a constant condition (25 °C, 60% RH). For each cycle, the corresponding adsorption time and the desorption time under simulated sunlight were 3 hours and 1.5 hours, respectively.

Indoor water harvesting at 20 °C, 60% RH. Dried PCl-4 (100 mg) was placed in a transparent container for water adsorption indoors. And a customized acrylic harvester (5 × 5 × 5 cm^3^) was used for water collecting. The device containing the above sorbent was exposed to simulated sunlight with 1 kW m^−2^ for water release and harvesting. During the testing, the temperature and RH were monitored by a temperature and humidity data logger.

### Characterization

The powder X-ray diffraction (XRD) data were collected on X-ray powder diffractometers (SmartLab, 5–50°, Cu Kα). Scanning electron microscopy (SEM) images were acquired using a Sigma 300. The SEM samples were prepared by loading the sample on a silicon wafer. The X-ray photoelectron spectroscopy (XPS) data were acquired with AXIS SUPRA, Kratas. The Fourier-transform infrared spectroscopy (FTIR) spectra were recorded from KBr pellets as background in the range 4000–400 cm^−1^ on a Nicolet Is5 (Thermo Fisher). Raman spectra were carried out on Laser confocal Raman spectrometer (Horiba Scientific LabRAM HR Evolution) excited with a 514 nm laser. Thermogravimetry analysis-differential scanning calorimetry (TG-DSC) was conducted on NETZSCH STA-2500 instrument with a heating rate of 10 °C min^−1^ under nitrogen atmosphere. UV-vis-NIR spectra were measured by a spectrometer (UV-3600) with an integrating sphere in the wavelength range of 2500 to 200 nm. N_2_ sorption isotherms were recorded on an American Mike ASAP 2460 instrument and the surface area was calculated *via* Barrett–Emmett–Teller (BET) mechanism. A Xenon lamp (CELPE300-3A) with a standard AM 1.5 G optical filter was used to simulate sunlight, and the optical intensity was measured with an optical power density meter (CEL-NP2000). Infrared camera (FLIR, E40) was used to record the photo-thermal data. Volumetric water adsorption isotherms are obtained on a BELSORP-MAXII instrument. Temperature and humidity were recorded on a data logger (COS-03) automatically.

## Results and discussion

### Fabrication and characterization of sorbents

**Fig. 1:**
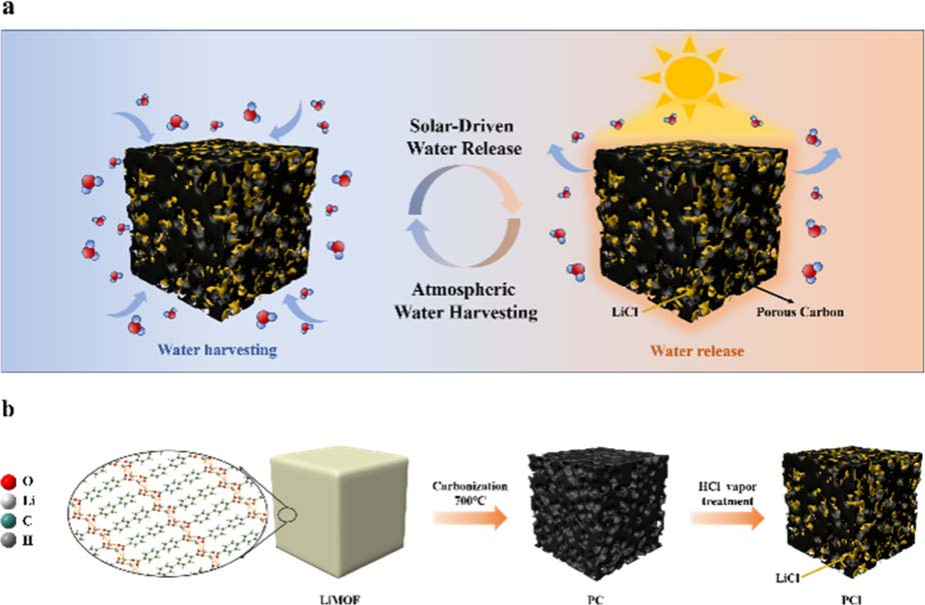
(a) Schematic illustration of PCl sorbents for atmospheric water harvesting and releasing under simulated sunlight. (b) Schematic illustration of the fabrication process of PCl.

**Fig. 2:**
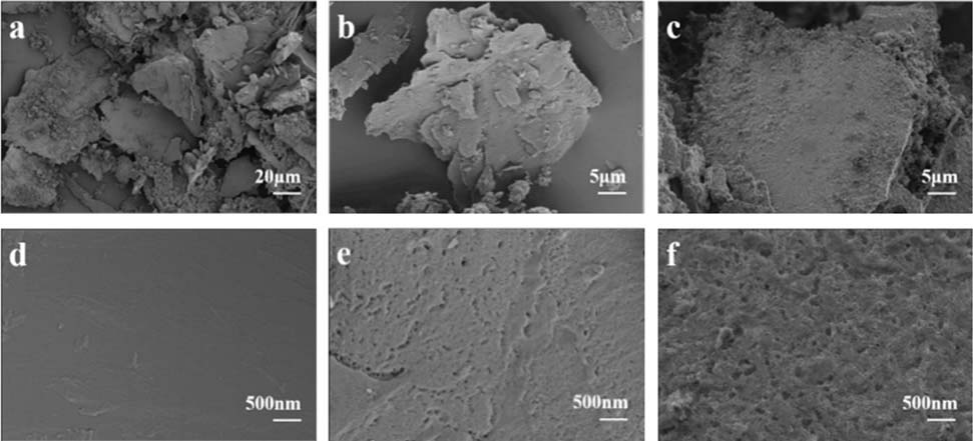
SEM images of LiMOF (a and d), PC-700 (b and e) and PCl-4 (c and f).

**Fig. 3:**
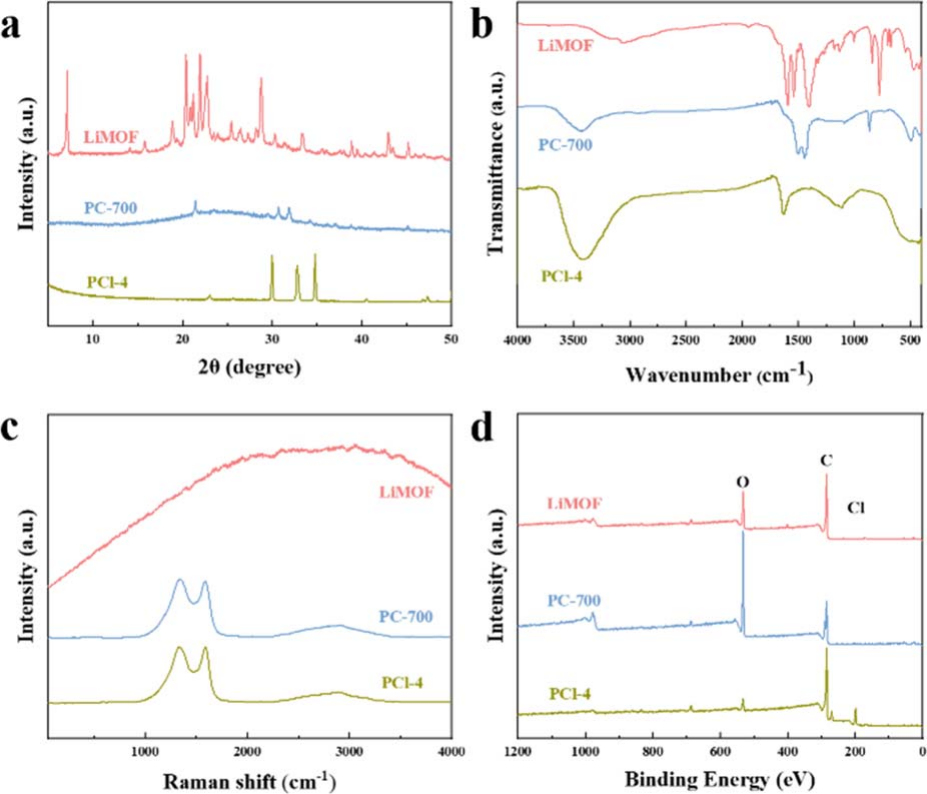
(a) XRD, (b) FTIR, (c) Raman spectra and (d) XPS spectra of LiMOF, PC-700, PCl-4, respectively.

Fig. 1b illustrates the fabrication process of PCl sorbents *via* the high temperature pyrolysis and HCl vapor treating methods. The temperature of pyrolysis is determined by TG-DSC results of LiMOF (Fig. S1, ESI†). As shown in the Fig. 2a and d, LiMOF is block-shaped, with a smooth and flat surface, and almost no pores. And the XRD patterns (Fig. 3a) display the successful synthesis of LiMOF as prevenient, all the characteristic peaks match well with those reported.^33^ Subsequently, LiMOF was subjected to carbonization at different temperatures to form PC, and as the carbonization temperature increased, there are noticeable morphological changes occurred on PC samples, and porous structure appeared on the surface (Fig. 2b, e and S2, ESI†). However, the characteristic XRD peaks of LiMOF gradually weakened, and the diffraction peaks of lithium carbonate (Li_2_CO_3_) appear (Fig. 3a and S3a, ESI†). PC-700 was obtained after high-temperature pyrolysis at 700 °C for 2 hours. Hierarchical pores appear on its surface, and there are only diffraction peaks of Li_2_CO_3_ in the XRD pattern (PDF#80-1307). The FTIR spectra (Fig. 3b and S4a, ESI†) also indicate that the absorption peaks at 1600 cm^−1^ and 1400 cm^−1^ corresponding to asymmetric stretching and symmetric stretching vibrations of the carboxylate groups of LiMOF weaken with carbonization temperature increasing, suggesting the partial bond cleavage in the MOF precursor.^34,35^ Meanwhile, the vibration of CO_3_^2−^ at 1500 cm^−1^ and 1440 cm^−1^ intensifies, and the peaks become sharp (Fig. S4a, ESI†). The characteristic peaks of PC-700 align with the characteristic peaks of Li_2_CO_3_ in the literature,^36^ confirming the formation of Li_2_CO_3_. The Raman spectrum of PC-700 exhibits broad bands at 1350 cm^−1^ and 1580 cm^−1^ in Fig. 3c, which are confirmed the generation of D-band (structural defect mode) and G-band (graphite mode). After pyrolysis, the graphitization degree of PC-700 increases compared to LiMOF. The intensity ratio, *I*_D_/*I*_G_, identifies the concentration of defects on the surface of the carbon materials. As shown in Fig. S5a (ESI),† the *I*_D_/*I*_G_ values of PC-500, PC-600, and PC-700 are 0.82, 0.96, and 1.04, respectively, which rise gradually as the increase of carbonization temperature, indicating the growth of defective carbon contents of PC. The defective carbon, which makes PC form more complex light-trapping structure, thus enhancing the reflection of light inside the material and improving the light absorbance.^37^

Then PC-700 was selected for the next step of HCl (36–38%) vapor treatment and the obtained product was named PCl. Table S1† shows the LiCl loading weight of different PCl samples. The SEM images show no significant morphological changes of PCl after HCl vapor treatment (Fig. 2c, f and S6, ESI†). The XRD patterns in Fig. 3a and S3b† exhibit characteristic peaks of LiCl (PDF#74-1181) at 30.09° ((111) lattice plane) and 34.88° ((200) lattice plane) appeared in all PCl samples with different treatment time, indicating the formation of LiCl. The diffraction peaks originating from lithium chloride hydrate (LiCl·H_2_O) at 23.17° ((200) lattice face), 32.89° ((202) lattice face) and 40.6° ((222) lattice face) also appears due to the strong moisture affinity of LiCl (PDF#22-1142). Meanwhile, the IR vibration of CO_3_^2−^ disappears in PCl-4 and PCl-12, indicating the CO_3_^2−^ groups are broken by HCl vapor (Fig. 3b and S4b, ESI†). The band ranging from 3200 cm^−1^ to 3500 cm^−1^ is due to the characteristic absorption of the O–H stretching vibrations of absorbed water.^38^ Among three samples, the higher intensity of the bands corresponding to hydrogen bonding of the PCl-4 sorbents, demonstrating a higher water sorption capacity of the PCl-4 than that of PC-700 (Fig. 3b). The XPS spectra of LiMOF, PC and PCl are shown in Fig. 3d and S7 (ESI).† After HCl treatment, the peak around 531 eV (O 1s) weaken, and the peaks around 270 eV (Cl 2s) and 198 eV (Cl 2p) appear in the spectrum of PCl, further proving that the CO_3_^2−^ was destroyed. The above results collectively demonstrate that the formed Li_2_CO_3_ is converted into LiCl after HCl treatment. Raman results show that increasing the time of HCl treatment has little effect on the structural defects of the samples (Fig. S5b, ESI†).

**Fig. 4:**
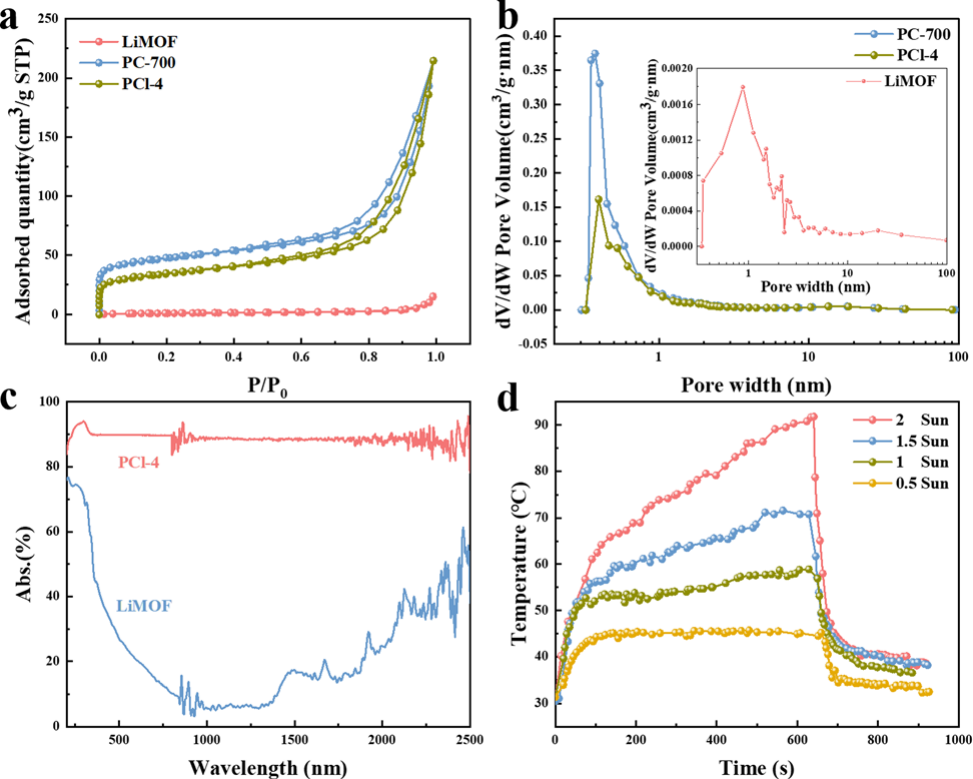
(a) The N_2_ adsorption–desorption isotherms and (b) pore size distribution curves of LiMOF, PC-700 and PCl-4. (c) UV-vis-NIR absorption spectra of LiMOF and PCl-4. (d) Temperature change of PCl-4 depend on time under different light intensities.

The N_2_ adsorption–desorption isotherms and pore size distribution of LiMOF show that there are almost no pores in LiMOF (Fig. 4a). Its BET surface area and pore volume are 4.60 m^2^ g^−1^ and 0.0235 cm^3^ g^−1^, respectively. In contrast, PCl-4 and PC-700 samples have a higher BET of 122.63 m^2^ g^−1^ and 173.20 m^2^ g^−1^, respectively, which is related to the porous structure formed after pyrolysis. Besides, PCl-4 and PC-700 samples both demonstrate a typical “type-IV”adsorption–desorption isotherm with a rapid rise at low pressure and vertical tails around the relative pressure to 1, indicating the coexistence of micro- and macropores.^39^ Additionally, the hysteresis loops observed in high-pressure zones are caused by the presence of mesopores in the samples.^40^ All of these indicate the formation of hierarchical porous structures. The pore volume of PCl-4 is 0.29 cm^3^ g^−1^, which is slightly smaller than 0.30 cm^3^ g^−1^ of PC-700. According to Fig. 4b, there are no obvious difference between PC-700 and PCl-4 in the range of pore width more than 1 nm, demonstrates that the pores in PC-700 are almost remained after HCl vapor treatment. The kinetic diameter of individual water molecule is 0.27–0.32 nm,^41,42^ thus water molecules can easily transport and diffuse within PCl-4. The distribution of all the micropores less than 1 nm (0.32–0.88 nm) declines after HCl vapor treatment, corresponding to the occupation of certain pore spaces by the interaction of Cl and Li. By contrast, immersing PCl-4 in water to attempt to remove LiCl, which results in a significant increase in both specific surface area (506.03 m^2^ g^−1^) and pore volume (0.73 cm^3^ g^−1^) for the obtained product PCl-4-w (Fig. S8a, ESI†). Additionally, it can be seen in Fig. S8b (ESI),† the corresponding pore size distribution curve shows that the pore sizes of PCl-4-w are enlarged compared to those of PCl-4. This demonstrates that the pyrolysis and HCl vapor treatment processes lead to the generation of hierarchical porous structures. Moreover, the MOF-derived porous carbon provides sufficient pore space for decorating LiCl and offers abundant adsorption sites and diffusion channels for water capture.

In order to investigate the photothermal properties of the material, light absorption and photothermal behaviors of PCl-4 samples were performed. The UV-vis-NIR spectrum reveals that PCl-4 has a good broadband solar absorptivity (∼90%) from 200 to 2500 nm as shown in Fig. 4c. Compared with LiMOF, the light absorbance of PCl-4 is greatly improved due to the well-maintained hierarchical porous structure of PC-700, which enhances the reflection of light inside the material and multiple scattering effects.^43,44^ The excellent photothermal property makes it possible to facilitate high efficiency of solar-to-thermal conversion. Besides, Fig. 4d shows the surface temperature changes of dry PCl-4 depend on time under different sunlight intensities. The surface temperature was monitored by an infrared (IR) camera. When the simulated light intensity is 1 kW m^−2^ (1 Sun), the surface temperature of dried PCl-4 can increase quickly from 31.1 °C to 53.5 °C in first 2 minutes and maintain a steady upward trend, reaching 58.9 °C after 10 minutes. When the light intensity increases, the surface temperature of PCl-4 can rise from room temperature (∼30 °C) to 70.8 and 90.8 °C within 10 minutes at 1.5 and 2 Suns, respectively. Then, PCl-4 cools down quickly within minutes when sunlight is removed. This fast photothermal response is attributed to the rapid heat transfer of the porous carbon, which is crucial for water releasing process under sunlight irradiation.

### Water adsorption–desorption performance

**Fig. 5:**
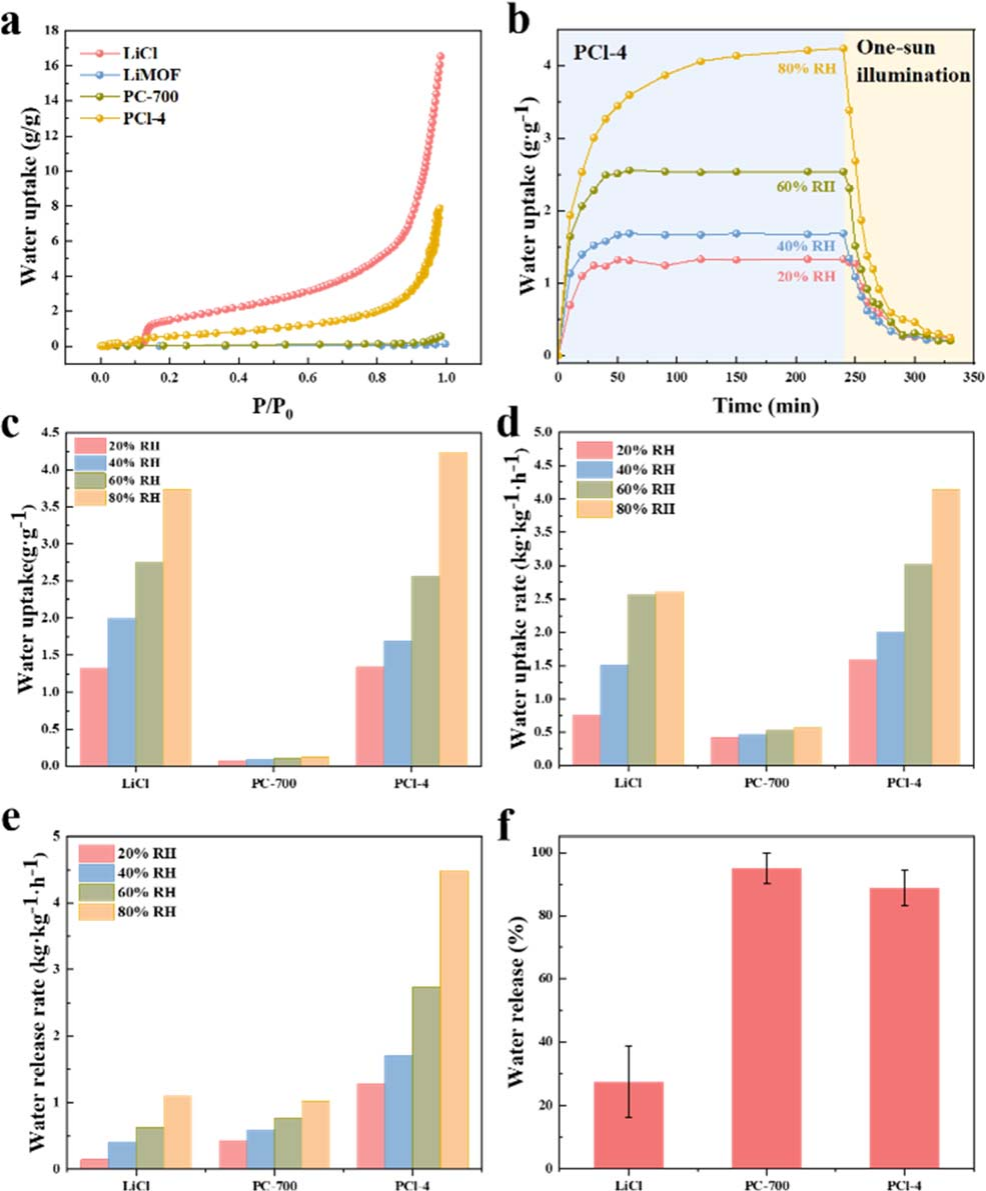
(a) Water vapor sorption performance of LiCl, LiMOF, PC-700 and PCl-4. (b) The calculated static water uptake curves of LiCl in PCl-4 at 20–80% RH. (c) The calculated water adsorption capacity of pure LiCl, PC-700 and LiCl in PCl-4 at 20–80% RH. (d) The calculated water uptake and (e) release rates of pure LiCl, PC-700 and LiCl in PCl-4. (f) Comparison of water release performance of pure LiCl, PC-700 and PCl-4.

The sorbents' performance is assessed based on two factors, its equilibrium water adsorption capacity and the kinetics of adsorption and desorption. Fig. 5a displays the water vapor adsorption isotherms of LiMOF, PC-700, PCl-4 and pure LiCl, illustrating the maximum adsorption capacity under different relative pressure. Obviously, LiMOF does not exhibit water adsorption capacity over a wide range of humidity levels. By contrast, the overall adsorption capacity of PCl-4 is significantly higher than that of PC-700, and the maximum adsorption capacity of PCl-4 (7.87 g g^−1^) is around 13.8 times that of PC-700 (0.57 g g^−1^), indicating that the LiCl decorated in porous sorbents plays a predominant role in water adsorption. However, pure LiCl shows much higher equilibrium water uptake than PCl-4 sorbents, which is due to the less amount of LiCl in PCl-4.

To further evaluate the water adsorption and release kinetics of all samples, static water adsorption tests were performed using a constant temperature and humidity cabinet under the conditions designed at 25 °C and 20–80% RH. The pre-dried samples were placed in a programmed instrument and exposed to specific conditions during the entire sorption test. And then solar simulator was turned on with one-sun illumination to study the desorption performance of the sorbents. The final results of the water uptake of different samples at 20–80% RH is shown in Fig. S9 (ESI).† When exposed to a specific RH (within 60% RH range), all PCl samples with different LiCl loading exhibit a rapid water adsorption within the initial 20 minutes (Fig. S9d–f, ESI†), followed by a gradual decrease in adsorption rate until reaching equilibrium in 60 minutes. However, LiCl and PCl sorbents did not reach equilibrium during sorption test at 80% RH. The water uptake of PCl-1, PCl-4 and PCl-12 samples is determined as 1.69, 1.66, 1.75 g g^−1^ at 80% RH after 240 minutes, respectively (Fig. S10b, ESI†). Compared with PC-700 (Fig. 5c), the adsorption capacity of these PCl (Fig. S10b, ESI†) is increased by about 14 times at 80% RH. This is mainly due to the fact that the porous carbon derived from LiMOF as precursor provides sufficient sites and pore space for the uniform distribution of LiCl in the structure. The *in situ* formed LiCl are the primary adsorption sites in extensive contact with water molecules, ultimately enhancing the overall adsorption capacity. The water uptake of PCl-12 is higher than that of PCl-1 and PCl-4, which is related to the more active sites provided by higher loading weight of LiCl in PCl-12. Additionally, the porous carbon framework with numerous structural defects also contributes to its adsorption capacity, which can be visualized in the static water uptake curves of PCl-4-W (Fig. S10a, ESI†).

With RH increasing from 20% to 40%, 60% and 80%, the water uptake (Fig. S10b, ESI†), water uptake rate (Fig. S10c, ESI†) and water release rate (Fig. S10d, ESI†) of the PCl sorbents increase accordingly. As for PCl-12, as RH increases from 20% to 80%, the water uptake and water uptake rate increase from 0.7 to 1.75 g g^−1^ and from 0.68 to 1.64 g g^−1^ h^−1^, respectively, indicating its wide working range. However, excess LiCl will limit its effectiveness in achieving higher water uptake. As PCl-12 has 1.25 times more LiCl content as compared to PCl-4, it only shows slightly higher water uptake and water uptake rate (Fig. S10, ESI†). In terms of desorption rate (Fig. S10d, ESI†), PCl-12 desorbs slower than PCl-4 at high humidity under one-sun illumination. And its water release performance of PCl-12 is also lower than that of PCl-4 (Fig. S10e, ESI†), which is due to the excessive LiCl loading making desorption difficult. As shown in Fig. 5e and S10d (ESI),† the water release rates of PCl-4 (Fig. S10d, ESI†) under simulated sunlight with an ambient humidity of 60% RH are 0.57, 0.70, 1.13, and 1.76 kg kg^−1^ h^−1^, significantly higher than that of LiCl (∼1.6–4.1 times). This is due to the outstanding photothermal performance of the MOF-derived porous adsorbents, serving as a solar absorber for efficient photothermal conversion. The unique pore structure of the prepared samples enhances the absorption of photon, which in turn increases the surface temperature of the sorbents *via* electron–phonon coupling, thus accelerates the solar-driven water desorption process. Though LiCl is impressive in water uptake, it suffers from a relatively high energy barrier in water desorption. The strong interaction between water and metal cations require higher regeneration temperature and longer drying time.^32^ In comparison, the PCl-4 sorbents display good water release performance (∼88%) within 1.5 hours of solar irradiation, while it is only ∼27% of water released in LiCl sample. In summary, porous sorbents decorated with LiCl can achieve more efficient solar water release without external energy input, aligning with the principles of sustainable development and environmental conservation.

Subsequently, the actual performance of LiCl in PCl samples are studied through calculations, and the results are shown in Fig. S11 (ESI).† The adsorption capacity and adsorption–desorption kinetics of LiCl in three PCl sorbents are compared, respectively. Firstly, it is obvious that the LiCl in PCl-4 has the highest water adsorption at 20%, 40%, 60% and 80% RH, which are 1.34, 1.69, 2.56 and 4.23 g g^−1^ respectively (Fig. S11c, ESI†). Although PCl-12 has the highest LiCl loadings, the LiCl in PCl-12 cannot be fully utilized and to capture water molecules effectively, resulting a lower performance. Furthermore, LiCl in PCl-4 exhibits an adsorption capacity closing to that of pure LiCl in Fig. 5c and a higher adsorption capacity at 80% RH compared to pure LiCl (3.74 g g^−1^), indicating the success of the porous carbon derived from LiMOF for outstanding water capacity. Hierarchical porous structure allows the evenly distribution of LiCl in PCl-4, which serves as adsorption sites to capture more water molecules. Fig. S11d and e† show that LiCl in PCl-4 has excellent water adsorption kinetics, with almost the highest water adsorption rate and water release rate in the range of 20–80% RH. Compared with pure LiCl (Fig. 5d and e), LiCl in PCl-4 has the fastest water collection and release capabilities. The water adsorption rates of LiCl in PCl-4 are 1.59, 2.00, 3.02, 4.13 kg kg^−1^ h^−1^, respectively, within the humidity range of 20–80% RH, which are about 1.2–2.1 times higher than that of pure LiCl. In particular, the calculated water release rates of LiCl in PCl-4 under one sun with different equilibrium loading are 1.28, 1.70, 2.73 and 4.48 kg kg^−1^ h^−1^, respectively (Fig. 5e), significantly better than those of pure LiCl (∼4.0–9.1 times). Simultaneously, the water release performance of LiCl in PCl-4 has been significantly enhanced, with a water release capability of 88.7% within 1.5 hours, far surpassing the 27% of pure LiCl (Fig. 5f). Generally, pure LiCl powder would turn into agglomerated crystals after several sorption cycles, and even lose the water sorption capacity mainly due to the worsened water transfer.^14^

However, the LiCl in PCl-4 exhibits much faster overall sorption/desorption kinetics than bulk LiCl during the water capture-release processes, which is related to the hierarchical porous matrix enhancing the heat and mass transfer, and the pore structures of the PCl sorbents providing sufficient adsorption space for water molecules and limited the size of LiCl nanocrystals. To realize the efficient utilization of LiCl, PCl-4 was selected and used in the following section for Practical water harvesting.

### Practical water harvesting demonstration

**Fig. 6:**
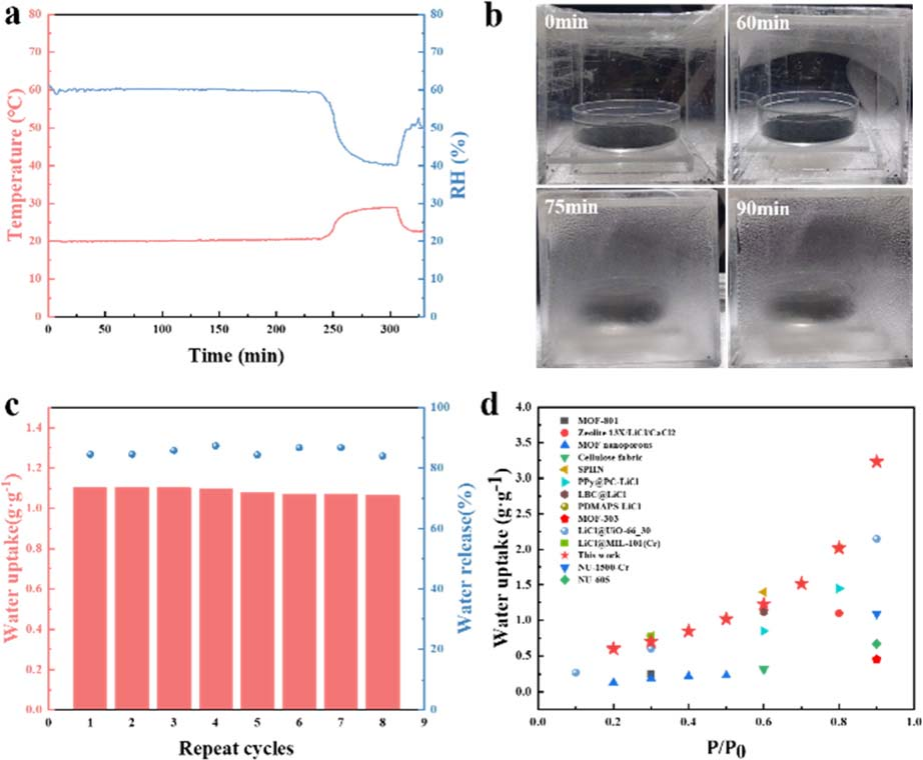
(a) The ambient temperature and humidity change curves and (b) optical images of the water harvester during the water release process. (c) The water sorption–desorption cycling tests of PCl-4 at 25 °C, 60% RH for sorption and then exposed to simulated sunlight at 1 kW m^−2^ for desorption. (d) The water adsorption capacity of most known adsorbents and PCl-4 in this work.

To assess the AWH performance of our sorbents in actual environment, practical water harvesting tests were conducted using a customized water harvester to showcase its feasibility. The water harvester was made of acrylic, a closed transparent cube container with a side length of approximately five centimeters. First, water harvesting experiment was conducted under ambient conditions (on Nov. 23, 2023, Wenzhou, China). The dried PCl-4 adsorbents were putted into an open container and then placed in the environment for a period of time to completely adsorb water vapor, followed by sealing it in the water harvester. The subsequent desorption process was performed by exposing the water harvester under simulated sunlight with an irradiance of 1 kW m^−2^. The water collection rate (%) is calculated as follows: *η* = *m*′/*m*′′, where *η* (%) is the water collection rate, *m*′ is the weight of collected water, and *m*′′ is the weight of released water. The corresponding ambient temperature and humidity change curves and optical images of the water harvester during the water release process are shown in Fig. 6a and b. The average temperature and RH was 20.4 °C and around 60% RH, respectively. Fig. 6b shows the released and condensed water on the inner wall of the harvester. Initially, the adsorbed water evaporated quickly, causing the transparent plastic cover to become foggy. As water continued evaporating, water vapor condensed on the inner wall of the container. The water uptake, release, and collection capacities of PCl-4 sorbents are 1.05, 0.92, and 0.83 g g^−1^, respectively, without the problem of salt leakage. In this condition, *η* is 90.2%.

In addition, PCl-4 sorbents were tested for stability and cyclic performance at 25 °C and 60% RH (Fig. 6c) by repeating the sorption/desorption process for eight times under 1 Sun. The water adsorption capacity of the first cycle is 1.10 g g^−1^ and its water release efficiency (%) is 84.5%. After eight sorption–desorption cycles, there was no obvious degradation in the performance of PCl-4, still remaining the same level (1.06 g g^−1^), and its water release performance fluctuates slightly relative to the first cyclic performance, indicating the excellent operational stability of PCl-4. In addition, the adsorption capacity at higher temperatures of PCl-4 was also tested (Fig. S12, ESI†). PCl-4 still maintains good adsorption performance at 35 °C and 45 °C, especially under high RH. At 20%, 40%, 60% and 80% RH, the adsorption capacities of PCl-4 at 45 °C are 0.38 g g^−1^, 0.60 g g^−1^, 0.98 g g^−1^, and 1.47 g g^−1^ respectively, maintaining 64%∼93% of the adsorption capacity at 25 °C. When the RH increases, such as above 60% RH, PCl-4 maintains more than 89% adsorption capacity compared to the adsorption capacity at 25 °C, indicating that the adaption and great potential of PCl-4 in extreme environment. Based on the above results, PCl-4 sorbents exhibit good stability and application potential, making it suitable for environmentally adaptive solar-driven AWH. We further compared the performance of PCl-4 sorbents with some representative sorbent materials, including MOFs, hygroscopic salt composites, *etc.* As shown in Fig. 6d, PCl-4 has outstanding water adsorption capacity in a wide range of RH, indicating that it is competitive with most adsorbents (Table S2, ESI†) and can serve as promising solar-powered AWH sorbents to collect water under various RH. Overall, this work provides a new and sustainable strategy for the development of MOFs-derived porous sorbents and may lead us further to harvest water from the atmosphere.

## Conclusions

In this work, we proposed a new design strategy in the way of using the Li-based MOF as a precursor material for pyrolysis, followed by HCl vapor treatment, to generate LiCl-decorated MOF-derived porous adsorbents (PCl-4) *in situ*. The sorbents combine the feature of high porosity and excellent photothermal properties of MOF-derived porous carbon, and present improved overall adsorption capacity and adsorption–desorption kinetics due to the stable and uniform distribution of LiCl, serving as adsorption sites in this porous structure. It reveals that LiCl in PCl-4 can be efficiently utilized, surpassing pure LiCl in both adsorption and desorption rates. The PCl-4, with efficient atmospheric water adsorption and rapid desorption capabilities, could collect 0.83 liter of water per kilogram of adsorbents within 5.5 hours under practical conditions. It exhibits good cyclic stability, maintaining excellent adsorption capacity and water release capabilities even after eight cycles. Therefore, this guides the design of porous materials with exceptional hygroscopicity and contributes to the advancement of solar-driven atmospheric water harvest technologies to tackle water scarcity in arid area.

## Author contributions

Simiao Guo: primary investigation, methodology, formal analysis, and original draft writing and editing. Yue Hu: writing, reviewing and editing. Zhou Fang: writing, reviewing and editing. Bing Yao: writing, reviewing and editing. Xinsheng Peng: conceptualization, supervision, reviewing and editing, resources, funding acquisition.

## Conflicts of interest

There are no conflicts to declare.

## Supplementary Material

RA-014-D4RA02364A-s001

## References

[cit1] Deng L. L., Guo S. L., Yin J. B., Zeng Y. J., Chen K. B. (2022). Sci. Rep..

[cit2] Sun S., Zheng X., Liu X., Wang Z., Liang L. (2022). Environ. Monit. Assess..

[cit3] Xie J., You J., Ma Z., Deng X., Lin P., Gao J. (2022). J. Cleaner Prod..

[cit4] Tashtoush B., Alshoubaki A. (2023). Energy.

[cit5] Li T., Wu M., Xu J., Du R., Yan T., Wang P., Bai Z., Wang R., Wang S. (2022). Nat. Commun..

[cit6] Ejeian M., Entezari A., Wang R. Z. (2020). Appl. Therm. Eng..

[cit7] Wang Q., Yang F., Wu D., Guo Z. (2023). Colloids Surf. A Physicochem. Eng. Asp..

[cit8] Xi Z., Li S., Yu L., Yan H., Chen M. (2022). ACS Appl. Mater. Interfaces.

[cit9] Xu J., Zhang J., Fu B., Song C., Shang W., Tao P., Deng T. (2020). ACS Appl. Mater. Interfaces.

[cit10] Ura D. P., Knapczyk-Korczak J., Szewczyk P. K., Sroczyk E. A., Busolo T., Marzec M. M., Bernasik A., Kar-Narayan S., Stachewicz U. (2021). ACS Nano.

[cit11] Azeem M., Noman M. T., Wiener J., Petru M., Louda P. (2020). Environ. Technol. Innov..

[cit12] Xu J., Li T., Chao J., Wu S., Yan T., Li W., Cao B., Wang R. (2020). Angew. Chem., Int. Ed..

[cit13] Kumar D., Tiwari A., Agarwal V., Srivastava K. (2022). Int. J. Environ. Sci. Technol..

[cit14] Lu K., Liu C., Liu J., He Y., Tian X., Liu Z., Cao Y., Shen Y., Huang W., Zhang K. (2022). ACS Appl. Mater. Interfaces.

[cit15] Li R., Shi Y., Wu M., Hong S., Wang P. (2020). Nano Energy.

[cit16] Wang W. W., Xie S. T., Pan Q. W., Dai Y. J., Wang R. Z., Ge T. S. (2021). Renew. Sust. Energ. Rev..

[cit17] Shan H., Poredos P., Ye Z., Qu H., Zhang Y., Zhou M., Wang R., Tan S. C. (2023). Adv. Mater..

[cit18] Xu J., Li T., Chao J., Wu S., Wang R. (2020). Angew. Chem., Int. Ed..

[cit19] Wasti T. Z., Sultan M., Aleem M., Sajjad U., Farooq M., Raza H. M. U., Khan M. U., Noor S. (2022). Adv. Mech. Eng..

[cit20] Ma A., Cong H., Deng H. (2022). Green Energy Environ..

[cit21] Xu H. J., Hu P. Y. (2023). J. Clean Prod..

[cit22] Kim S. Y. H., Rao S. R., Narayanan S., Kapustin E. A., Furukawa H., Umans A. S., Yaghi O. M., Wang E. N. (2017). Science.

[cit23] Hanikel N., Prevot M. S., Yaghi O. M. (2020). Nat. Nanotechnol..

[cit24] Hu Y., Wang Y., Fang Z., Yao B., Ye Z., Peng X. (2023). ACS Appl. Mater. Interfaces.

[cit25] Song Y., Xu N., Liu G., Qi H., Zhao W., Zhu B., Zhou L., Zhu J. (2022). Nat. Nanotechnol..

[cit26] Ying Y., Yang G., Tao Y., Wu Q., Li H. (2023). Adv. Sci..

[cit27] Graeber G., Diaz-Marin C. D., Gaugler L. C., Zhong Y., El Fil B., Liu X., Wang E. N. (2023). Adv. Mater..

[cit28] Aleid S., Wu M., Li R., Wang W., Zhang C., Zhang L., Wang P. (2022). ACS Mater. Lett..

[cit29] Sun Y., Spieß A., Jansen C., Nuhnen A., Gökpinar S., Wiedey R., Ernst S. J., Janiak C. (2020). J. Mater. Chem. A.

[cit30] Dang S., Zhu Q. L., Xu Q. (2017). Nat. Rev. Mater..

[cit31] Chen G., Jiang Z., Li A., Chen X., Ma Z., Song H. (2022). ACS Sustain. Chem. Eng..

[cit32] Lin H., Yang Y., Hsu Y. C., Zhang J., Welton C., Afolabi I., Loo M., Zhou H. C. (2024). Adv. Mater..

[cit33] Banerjee D., Borkowski L., Kim S., Parise J. (2009). Cryst. Growth Des..

[cit34] Sabet M., Tanreh S., Khosravi A., Astaraki M., Rezvani M., Darvish Ganji M. (2022). Diamond Relat. Mater..

[cit35] Luo R., Xu H., Gu H., Wang X., Xu Y., Shen X., Bao W., Zhu D. (2014). Cryst. Eng. Comm..

[cit36] Chang X., Fan M., Yuan B., Gu C. F., He W. H., Li C., Feng X. X., Xin S., Meng Q., Wan L. J., Guo Y. G. (2023). Angew. Chem., Int. Ed..

[cit37] Tang Y. L., Zhao X. G., Li D. K., Zuo X. C., Tang A. D., Yang H. M. (2022). Sol. Energy Mater. Sol. Cells.

[cit38] Chen B., Jing S., Chen Q., Pei Y., Deng T., Yang B., Wang C., Li T. (2023). Nano Energy.

[cit39] Fang Z., Deng Z., Wan X., Li Z., Ma X., Hussain S., Ye Z., Peng X. (2021). Appl. Catal., B.

[cit40] Zhang X., Li Q., Fan M., Xu G., Liu X., Gong H., Deng J., Meng S., Wang C., Wang Z., Wei Y., Liu J., Peng Y. (2022). J. CO2 Util..

[cit41] Li A., Xiong J., Liu Y., Wang L., Qin X., Yu J. (2022). Energy Environ. Mater..

[cit42] Zhang Y., Wu L., Wang X., Yu J., Ding B. (2020). Nat. Commun..

[cit43] Yang X., Chen L., Li Y., Rooke J. C., Sanchez C., Su B. (2017). Chem. Soc. Rev..

[cit44] Li Y., Fu Z. Y., Su B. L. (2012). Adv. Funct. Mater..

