# Supplementary material for: LiCl in situ decorated metal–organic framework (MOF)-derived porous carbon for efficient solar-driven atmospheric water harvesting
Source: RSC Adv. 2024 May 14;14(22):15619–26. doi: 10.1039/d4ra02364a (PMC11091862; doi:10.1039/d4ra02364a)
Supplement: RA-014-D4RA02364A-s001 [file RA-014-D4RA02364A-s001.pdf]

Electronic Supplementary Material (ESI) for RSC Advances.
This journal is © The Royal Society of Chemistry 2024

Electronic Supplementary Information

LiCl in-situ decorated metal-organic framework (MOF)-Derived Porous Carbon for Efficient Solar-Driven Atmospheric Water Harvesting

Simiao Guo,[a, b] Yue Hu,[a, b] Zhou Fang,[a, b] Bing Yao,[a, b] and Xinsheng Peng[a,b*]

[a]State Key Laboratory of Silicon and Advanced Semiconductor Materials, School of Materials Science and Engineering, Zhejiang University, Hangzhou 310058, P. R. China

[b]Wenzhou Key Laboratory of Novel Optoelectronic and Nanomaterials, Institute of Wenzhou, Zhejiang University, Wenzhou 325006, P. R. China

E-mail: pengxinsheng@zju.edu.cn

The file includes:

1. **Equation S1-S8.**

2. **Figures S1-S12.**

3. **Tables S1-S2.**

S1. Calculations to evaluate adsorbent performance

The water adsorption, desorption, and collection capacity (g g^{-1}) were used to evaluate the AWH performance of the sorbents. And the corresponding water uptake and release rates (kg kg^{-1} h^{-1}) were calculated to compare their sorption-desorption kinetics. They were calculated by the following equations:

$$\text{Water adsorption} = (W_1 - W_s) / W_s \quad \text{(S1)}$$

$$\text{Water desorption} = (W_1 - W_2) / W_s \quad \text{(S2)}$$

$$\text{Water collection} = W_3 / W_s \quad \text{(S3)}$$

$$\text{Water adsorption rates} = (W_{t1} - W_s) / (W_s \times t_1) \quad \text{(S4)}$$

$$\text{Water desorption rates} = (W_1 - W_{t2}) / (W_s \times t_2) \quad \text{(S5)}$$

Where Ws (g) is the weight of the dried sorbents before adsorption tests, W_1 (g) is the weight of the sorbent corresponding to the maximum adsorption value at a certain moment in the adsorption test, W_2 (g) stands for the weight of the samples after desorption for a certain period of time, W_3 (g) is the weight of the collected water after desorption and condensation; t_1 (hours) is the sorption time corresponding to the inflection point before water adsorption reaching equilibrium, t_1 is chosen to be ~0.833 hours in this work, W_{t1} (g) is the weight of sorbents corresponding to t_1, t_2 (hours) is the desorption time corresponding to the inflection point before water desorption reaching equilibrium, t_2 is chosen to be ~0.833 hours in this work; W_{t2} (g) is the weight of sorbents corresponding to t_2.

As for the performance of LiCl in PCl sorbents, the relevant formulas are as follows:

$$\text{Water adsorption} = Q = (Q_{PCl} - Q_{PCl\text{-}w} \times C_{PCl\text{-}w}) / C_{LiCl} \quad \text{(S6)}$$

$$\text{Water adsorption rates} = Q_{t1} / t_1 \quad \text{(S7)}$$

$$\text{Water desorption rates} = (Q_m - Q_{t2}) / t_2 \quad \text{(S8)}$$

Where Q_{PCl} (g g^{-1}) is the water adsorption capacity of PCl sorbents, $Q_{PCl\text{-}w}$ (g g^{-1}) is the water adsorption capacity of PCl-w, C_{LiCl} (wt.%) represents the loading weight of LiCl in PCl, while $C_{PCl\text{-}w}$ (wt. %) represents the loading weight of porous carbon after removing LiCl ($C_{PCl\text{-}w} + C_{LiCl} = 1$); Q_{t1} (g g^{-1}) is the water adsorption of LiCl in PCl sorbents corresponding to t_1, Q_m (g g^{-1}) is the water adsorption of LiCl in PCl when the

reaching water adsorption equilibrium, Q_{t2} (g g^{-1}) is the weight of LiCl in PCl sorbents corresponding to t_2.

S2. Supporting Figures and Tables

Table S1. The amount of LiCl in PCl sorbents according to weighing method.

Sample	The amount of LiCl (wt. %)
PCl-1	38
PCl-4	36
PCl-12	45

Note: The amount of LiCl is calculated by weighing the mass change of adsorbents before and after HCl treatment.

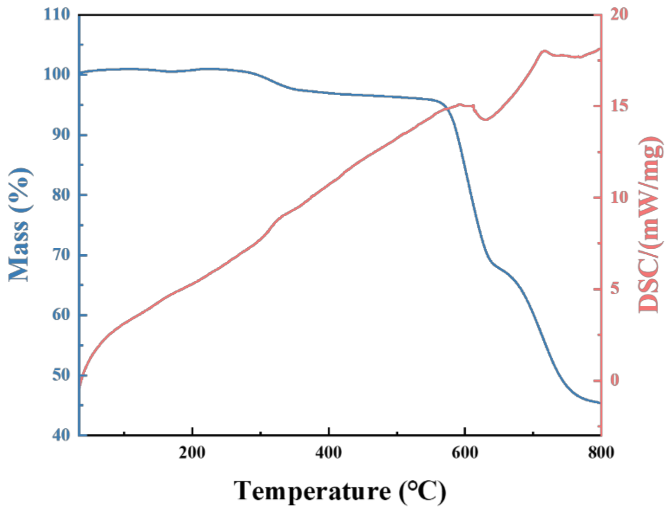

Fig. S1. The TG-DSC curves of LiMOF.

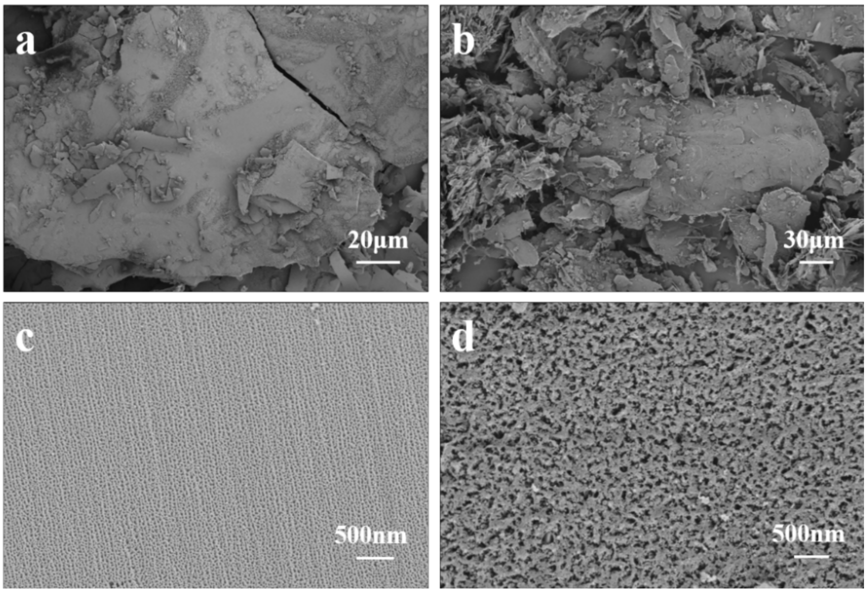

Fig. S2. SEM images of PC-500 (a, c) and PC-600 (b, d).

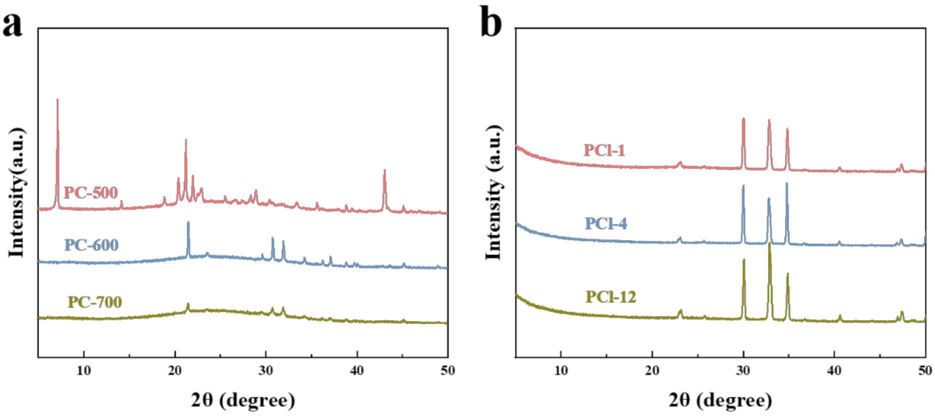

Fig. S3. XRD curves of the samples (a) synthesized at different carbonization temperatures and (b) samples with different HCl vapor treatment times.

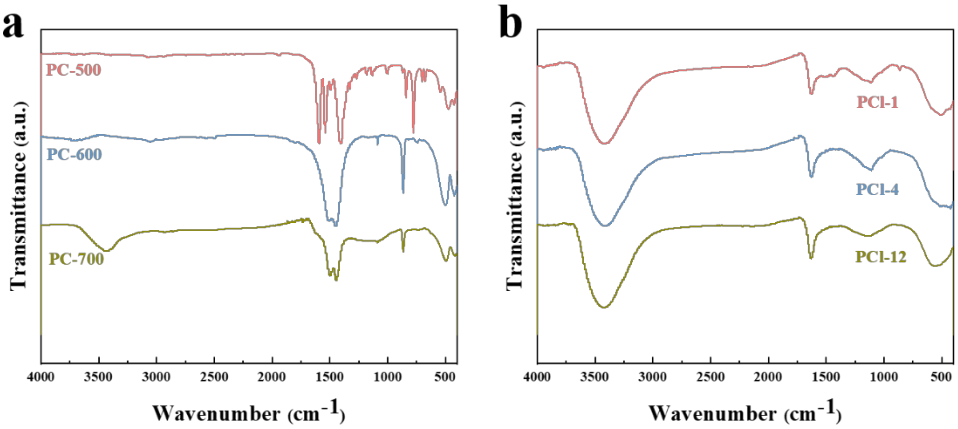

Fig. S4. FTIR spectra of the samples (a) synthesized at different carbonization temperatures and (b) samples with different HCl vapor treatment times.

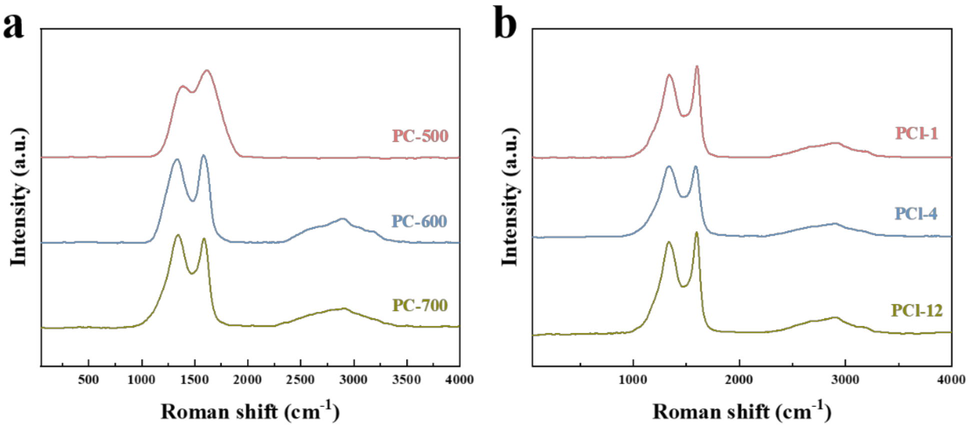

Fig. S5. Roman spectra of the samples (a) synthesized at different carbonization temperatures and (b)samples with different HCl vapor treatment times.

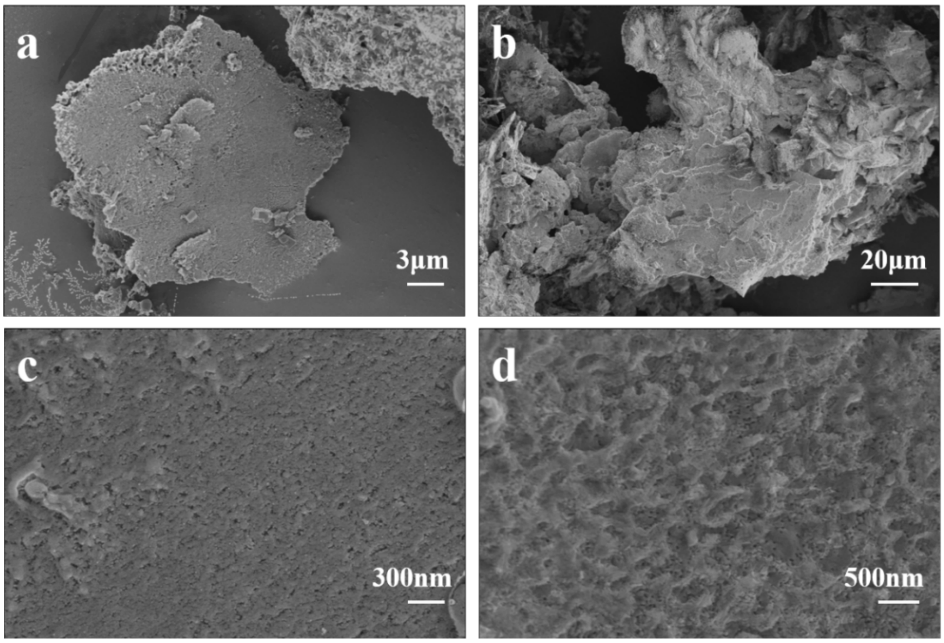

Fig. S6. SEM images of PCl-1 (a, c) and PCl-12 (b, d).

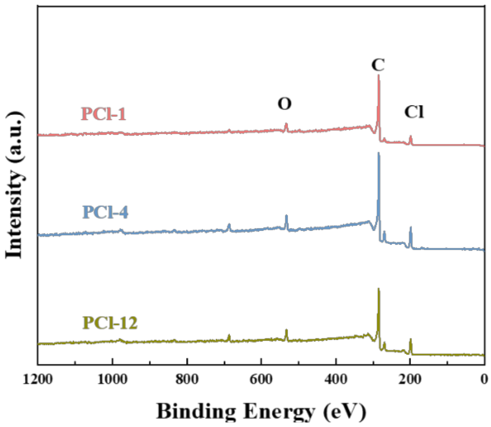

Fig. S7. XPS spectra of the samples with different HCl vapor treatment times.

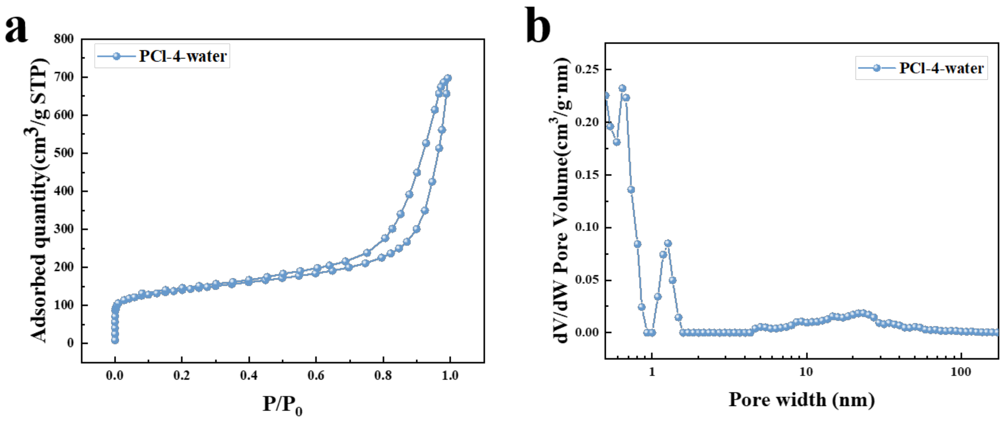

Fig. S8. (a) The N_2 adsorption-desorption isotherms and (b) pore size distribution curves of PCl-4-water.

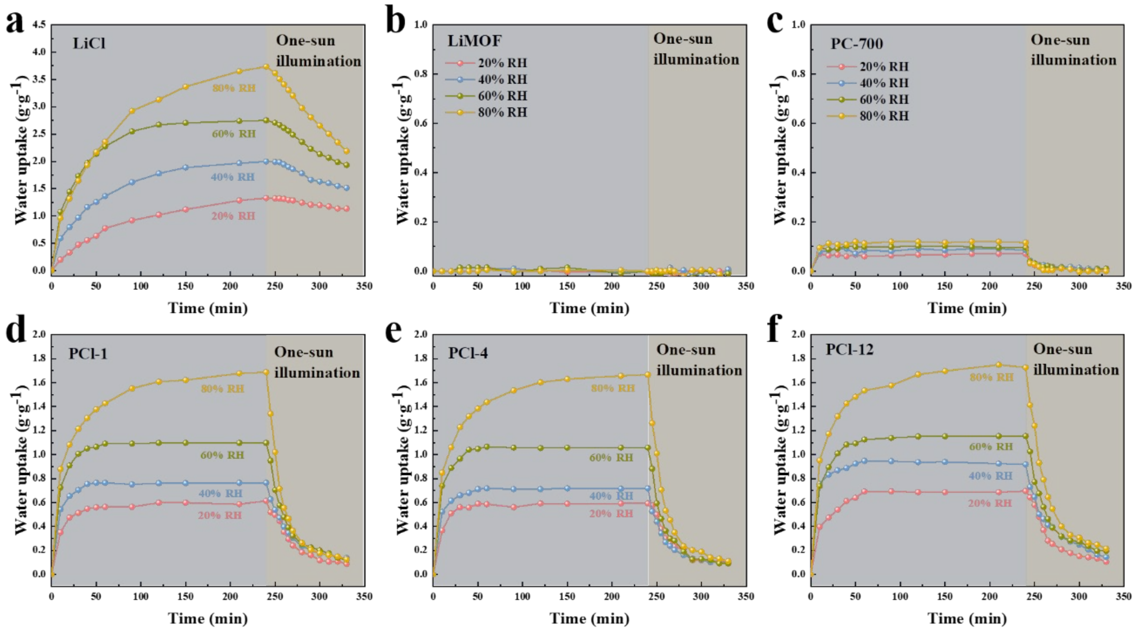

Fig. S9. Water uptake curves of (a) LiCl, (b) LiMOF, (c) PC-700, (d) PCl-1, (e) PCl-4, (f) PCl-12.

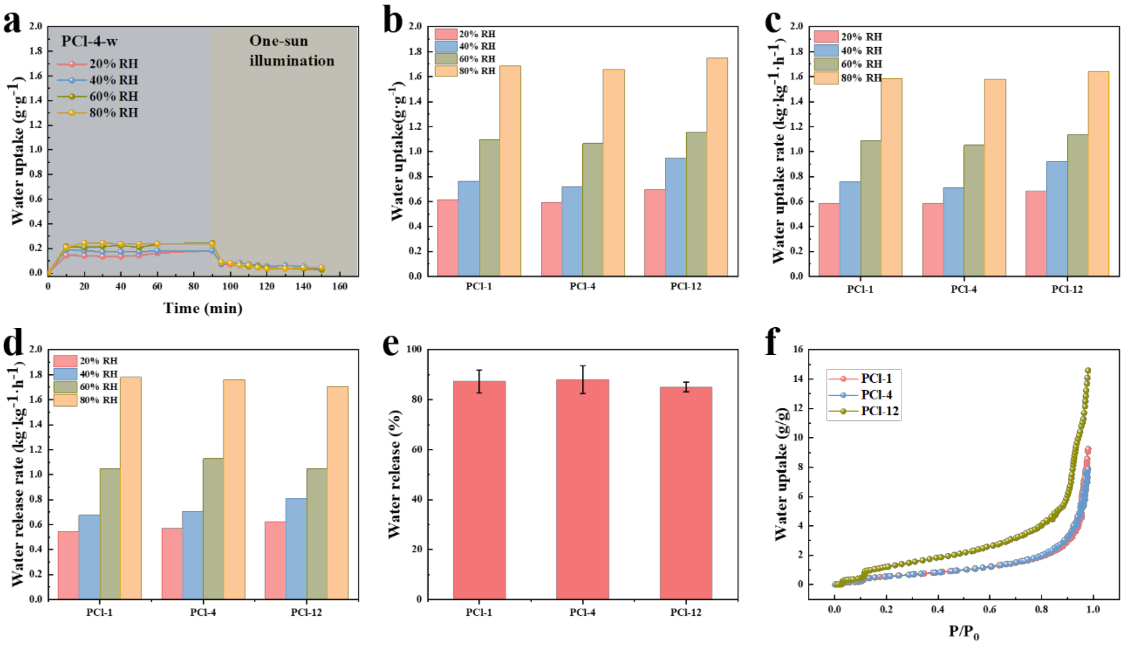

Fig. S10. (a) Static water uptake curves of PCl-4-w at 20-80% RH. (b) Water adsorption capacity at 20-80% RH. (c) The calculated water uptake and (d) release rates. (e) Comparison of water release performance. (f) Water vapor sorption performance of PCl-1, PCl-4, PCl-12.

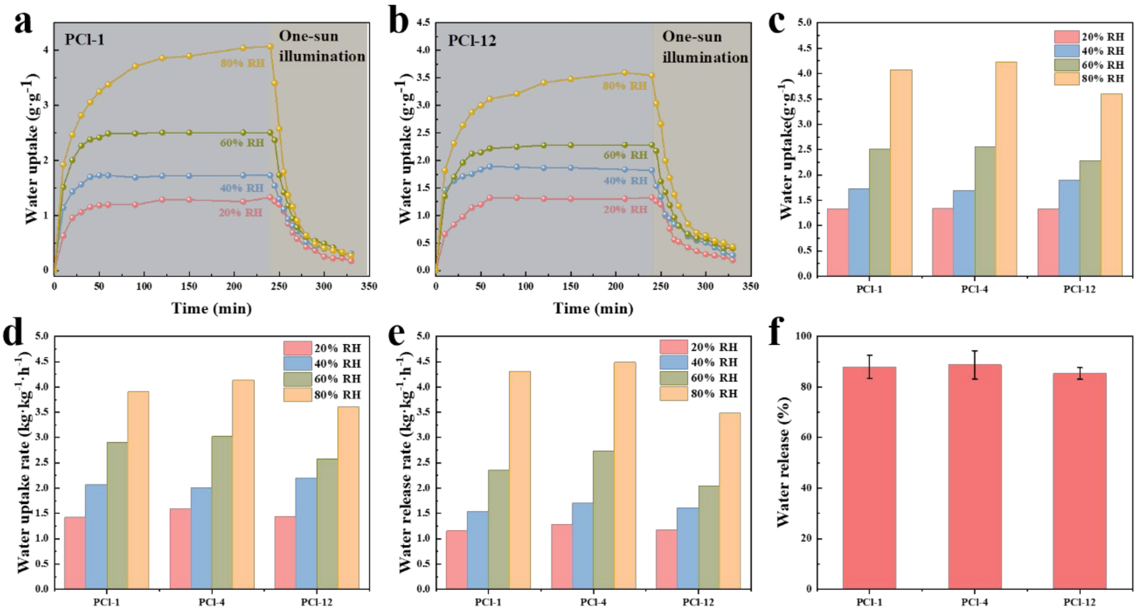

Fig. S11. Static water uptake curves of LiCl in PCl-1 (a) and LiCl in PCl-12 (b) at 20-80% RH. (c) Water adsorption capacity of LiCl in PCl at 20-80% RH. (d) The calculated water uptake and (e) release rates of LiCl in PCl. (f) Comparison of water release performance of LiCl in PCl.

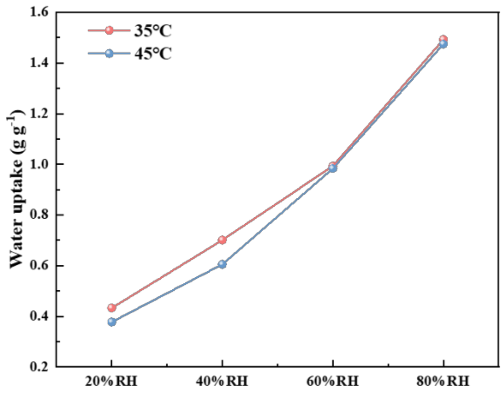

Fig. S12 Water adsorption capacity of PCl-4 at 35 °C and 45 °C.

Table S2. Comparison of the water sorption performance for the sorbents in this work and previous publications.

Materials	Temperature (°C)	Relative humidity (%)	Water uptake ($g\ g^{-1}$)	Ref.
MOF-801	25	30	0.25	[1]
Zeolite 13X/LiCl/$CaCl_2$	25	80	1.10	[2]
MOF nanoporous carbon	25	20	0.13	[3]
MOF nanoporous carbon	25	30	0.19	[3]
MOF nanoporous carbon	25	40	0.22	[3]
MOF nanoporous carbon	25	50	0.23	[3]
Cellulose fabric	25	60	0.31	[4]
SPHN	25	30	0.78	[5]
SPHN	25	60	1.4	[5]
SPHN	25	80	2.01	[5]
PPy@PC-LiCl	25	60	0.85	[6]
PPy@PC-LiCl	25	80	1.45	[6]
LBC@LiCl	25	60	1.12	[7]
PDMAPS-LiCl	25	60	0.62	[8]
MOF-303	25	90	0.45	[9]
LiCl@UiO-66_30	25	10	0.27	[10]
LiCl@UiO-66_30	25	30	0.27	[10]
LiCl@UiO-66_30	25	90	2.15	[10]
LiCl@MIL-101(Cr)	30	30	0.77	[11]
NU-1500-Cr	25	90	1.09	[12]
NU-605	25	90	0.67	[13]
PCl-4	25	20	0.6	Our work
PCl-4	25	40	0.85	Our work
PCl-4	25	60	1.23	Our work
PCl-4	25	80	2.02	Our work

References

1 H. Kim, S. R. Rao, E. A. Kapustin, L. Zhao, S. Yang, O. M. Yaghi and E. N. Wang, *Nat. Commun.*, 2018, **9**, 1191.

2 H. Zhao, Z. Wang, Q. Li, T. Wu, M. Zhang and Q. Shi, *Micropor. Mesopor. Mat.*, 2020, **299**, 110109.

3 Y. Song, N. Xu, G. Liu, H. Qi, W. Zhao, B. Zhu, L. Zhou and J. Zhu, *Nat. Nanotechnol.*, 2022, **17**, 857-863.

4 Y. Zhang, W. Zhu, C. Zhang, J. Peoples, X. Li, A. L. Felicelli, X. Shan, D. M. Warsinger, T. Borca-Tasciuc, X. Ruan and T. Li, *Nano Lett.*, 2022, **22**, 2618-2626.

5 X. Han, L. Zhong, L. Zhang, L. Zhu, M. Zhou, S. Wang, D. Yu, H. Chen, Y. Hou and Y. Zheng, *Small*, 2023, **19**, 2303358.

6 K. Lu, C. Liu, J. Liu, Y. He, X. Tian, Z. Liu, Y. Cao, Y. Shen, W. Huang and K. Zhang, *ACS Appl. Mater. Interfaces*, 2022, **14**, 33032-33040.

7 W. Yao, X. Zhu, Z. Xu, R. A. Davis, G. Liu, H. Zhong, X. Lin, P. Dong, M. Ye and J. Shen, *ACS Appl. Mater. Interfaces*, 2022, **14**, 4680-4689.

8 C. Lei, Y. Guo, W. Guan, H. Lu, W. Shi and G. Yu, *Angew. Chem. Int. Ed.*, 2022, **61**, e202200271.

9 X. K. P. N. Hanikel, S. Chheda, H. Lyu, W.S. Jeong, J. Sauer, L. Gagliardi, O. M. Yagh, *Science*, 2021, **374**, 454-459.

10 Y. Sun, A. Spieß, C. Jansen, A. Nuhnen, S. Gökpinar, R. Wiedey, S. J. Ernst and C. Janiak, *J. Mater. Chem. A.*, 2020, **8**, 13364-13375.

11 J. Xu, T. Li, J. Chao, S. Wu, T. Yan, W. Li, B. Cao and R. Wang, *Angew. Chem. Int. Ed.*, 2020, **59**, 5202-5210.

12 Z. Chen, P. Li, X. Zhang, P. Li, M. C. Wasson, T. Islamoglu, J. F. Stoddart and O. K. Farha, *J. Am. Chem. Soc.*, 2019, **141**, 2900-2905.

13 Y. Chen, K. B. Idrees, M. R. Mian, F. A. Son, C. Zhang, X. Wang and O. K. Farha, *J. Am. Chem. Soc.*, 2023, **145**, 3055-3063.
